# The Mechanisms of Lithium Action: The Old and New Findings

**DOI:** 10.3390/ph18040467

**Published:** 2025-03-26

**Authors:** Kosma Sakrajda, Janusz K. Rybakowski

**Affiliations:** 1 Molecular and Cell Biology Unit, Poznan University of Medical Sciences, 60-572 Poznan, Poland; 2 Department of Adult Psychiatry, Poznan University of Medical Sciences, 60-572 Poznan, Poland

**Keywords:** lithium, bipolar disorder, lithium response

## Abstract

Despite lithium’s presence in modern psychiatry for three-quarters of a century, the mechanisms of its therapeutic action have not been fully elucidated. This article presents the evolution of the views on these mechanisms, and both the old and new findings are discussed. Among the old mechanisms, lithium’s effect on the purinergic system; electrolyte metabolism; membrane transport; and second messenger systems, namely, cyclic nucleotide and phosphatidylinositol (PI), glycogen synthase kinase-3beta (GSK-3β), brain-derived neurotrophic factor, and neurotransmitters, are discussed. The new data were obtained from in vitro studies, molecular biology, and genetic research. They showed the effects of lithium on the immune system, biological rhythms, telomere functions, and mitochondria. In this article, each lithium mechanism is considered in the light of its association with the pathogenesis of bipolar disorder or/and as a marker of the lithium response. Although not exhaustive, this review elucidates the multiple potential mechanisms of lithium action. It was also observed that many seemingly “old” mechanisms have experienced a resurgence in research conducted during the 21st century. Additionally, many studies converged on the previously postulated mechanisms of lithium inhibiting GSK-3β and PI.

## 1. Introduction

In 2024, the 75th anniversary of introducing lithium into contemporary psychiatry was commemorated. This event was attested by an Australian psychiatrist John Cade’s article in the 1949 issue of the *Medical Journal of Australia* [1]. Furthermore, in 1963, the first observations appeared pointing to lithium’s property to prevent the recurrences of mood disorders [2]. Consequently, lithium is now the first-choice drug for preventing recurrences in mood disorders, mainly bipolar disorder (BD), as recommended in all major guidelines [3]. When treating an acute episode, lithium possesses strong antimanic and moderate antidepressant activity but is especially useful for the augmentation of antidepressant medications in treatment-resistant depression [4]. It was also demonstrated that lithium administration exerts suicide-preventive, immunomodulatory, and anti-dementia effects [5,6].

However, despite lithium’s presence in modern psychiatry for three-quarters of a century, the exact mechanisms of its therapeutic action still have not been fully elucidated. In this paper, we present the evolution of perspectives on various selected mechanisms, discussing both the historical and recent findings and highlighting the pivotal roles played by several investigators in certain discoveries. Each mechanism is considered in light of its association with the pathogenesis of BD or/and as a marker of the lithium response.

## 2. Old Findings

### 2.1. Uric Acid and Purinergic System

#### 2.1.1. Uric Acid

The influence of lithium treatment on uric acid levels, a crucial component of the purinergic system, can be regarded as the earliest proposed mechanism of its therapeutic action [7]. The first medical application of lithium was performed in 1859 by Alfred Garrod (1819–1907) to treat gout [8]. The rationale for using lithium was the previous observation of increased urate levels in patients with gout and the good solubility of the lithium urate. Therefore, lithium was meant to reduce the urates as lithium urate [8]. In the same context, in 1886, the Danish scientist Carl Lange (1834–1900) proposed uric acid diathesis as a pathogenic mechanism of periodical depression and suggested the therapeutic use of lithium carbonate as a treatment that allegedly normalizes the uric acid levels [9].

The concept of the pathogenic role of uric acid concerning mood disorders and lithium treatment also laid the foundation for the introduction of lithium to contemporary psychiatry by John Cade (1912–1980), who observed that the urine of manic patients was particularly toxic to guinea pigs. Cade attributed this to the excess of urates and administered lithium urate to the animals, obtaining a sedative effect, and subsequently also administered lithium carbonate with the same result. This effect further led him to treat manic patients with lithium carbonate with spectacular outcomes [1].

The observations of uric acid alterations in mood disorders might indicate the involvement of this substance in the pathogenesis of mood disorders and the effect of lithium. In 1968, it was shown that lithium had a uricosuric effect in patients with BD, and its excretion increased in the early phase of remission [10]. The 21st century brought further indications of uric acid’s role in the pathogenesis of bipolar disorder. Taiwanese researchers showed that 16.4% of patients with bipolar disorder and 13.6% of the control group had gout. The risk of onset of gout during the 6-year observation period was 1.19 times greater for people with BD than for the control group [11]. Further clinical studies revealed that patients with a first episode of mania had increased concentrations of uric acid, which decreased as the clinical condition improved after one month of treatment [12,13]. However, the 2016 meta-analysis conducted by Bartoli et al. showed that patients with BD had significantly higher serum concentrations of uric acid as compared with healthy individuals and those suffering from periodic depression [14]. Interestingly, the study by Muti et al. showed that lithium-treated patients’ serum lithium levels positively correlated with their uric acid levels [15]. Therefore, based on this result, the exact effect of lithium treatment on uric acid levels and the precise role of uric acid in BD should be further studied.

#### 2.1.2. Purinergic System

Lithium might also influence the purinergic signaling system. The concept of this system was proposed in the 1970s by Geoffrey Burnstock (1929–2020), where the purine nucleotides adenosine 5′-triphosphate (ATP) and adenosine diphosphate (ADP) were defined as extracellular messengers [16].

Gubert et al. showed that in patients with BD, the adenosine concentration was lower compared with the control group, and they also demonstrated the negative correlation between the adenosine concentration and depression severity [17]. An experimental study also found that lithium increases the adenosine concentration by inhibiting ectonucleotidase [18].

The purinergic receptor P2X7 role was previously postulated in the pathogenesis of BD. This receptor mediates the processes of apoptosis and proliferation, mediates the release of proinflammatory cytokines, and is also involved in the mechanisms of neurotransmission and neuromodulation [19]. The P2X7 receptor gene is located on chromosome 12q23-24, which is described as a potential locus responsible for BD. An association between the gene polymorphism of that receptor and a predisposition to BD has also been described [20].

Lithium’s effect on purinergic signaling can also be connected with the neuroprotective potential of the drug. Chronic lithium treatment inhibits ATP-induced cellular death by decreasing the ATP and increasing the adenosine levels [18]. The neuronal and microglial cells are responsive to in vitro ATP treatment, leading to ATP-mediated neural cell death and microglia phenotypes that respond to an inflammatory state. Lithium treatment results in the modulation of neuronal cell response by preventing cell death; however, it cannot prevent the microglia inflammatory response. This effect leads to the conclusion that the purinergic-based mechanism of lithium action is more likely to occur in neurons [21], but the exact purinergic-based neuroprotective effect should be further studied.

### 2.2. Electrolyte Metabolism and Membrane Transport

Lithium is a monovalent cation from the first group of the Mendeleev table, along with sodium and potassium. In the 1960s/1970s, interest was kindled in the possible effect of lithium on electrolyte metabolism and transmembrane transport in relation to its therapeutic mechanisms.

#### 2.2.1. Lithium Accumulation in the Organism

An Australian psychiatrist, Maurice Serry, hypothesized that the therapeutic response to lithium can be proportional to the amount of retention of this ion in the organism. He elaborated on the lithium excretion test measuring the excretion of lithium within 4 h after loading a dose of 1200 mg of lithium carbonate [22]. He found that manic patients who were lithium retainers responded to lithium favorably, while the subjects that excessively excreted lithium did not [23]. It is considerable that the higher retention of lithium could be directly related to a decreased elimination of lithium from the cytosol, which is the topic of the following subsection.

#### 2.2.2. Transmembrane Lithium Transport

In 1975, the primary mechanism of transporting lithium out of the cells by the lithium–sodium counter-transport system (LSC) was discovered. This discovery was made based on red blood cells by a team at Harvard Medical School led by Daniel Tosteson (1925–2009), the dean of the school from 1977 to 1997 [24]. Since the LSC determines the lithium concentration in some cells, e.g., in erythrocytes, its activity is related to the red blood cell lithium ratio. This index was further widely investigated concerning the pathogenesis of BD and therapeutic aspects of lithium treatment. A higher lithium blood cell index determines the ratio of lithium concentration in cells to that in serum. In patients with bipolar affective disorder, a higher concentration of lithium in the red blood cell was found in comparison with healthy subjects. This observation could indicate a reduced efflux of lithium from the blood cells governed by the LSC [25,26]. In other studies, the genetic component of the lithium ratio was established [27] and a lower rate of lithium transport from the cells in bipolar patients compared with control subjects was confirmed [28]. Unfortunately, the direct potential of the LSC mechanism for the pathogenesis of BD and the quality of lithium response has not been investigated in subsequent decades. However, the recent systematic review by Coyac et al. [29] of studies that researched the red blood cell lithium concentration and red blood cell/plasma lithium ratio in mood disorders concluded that further studies on these factors could be helpful as a novel method for monitoring lithium treatment tolerance.

#### 2.2.3. Sodium–Potassium ATPase

In the 1970s/1980s, the findings of the decreased activity of the sodium–potassium-activated adenosine triphosphate pump (sodium–potassium ATPase) in erythrocytes of mood-disorder-suffering patients were reported, as well as the increased activity of this pump after lithium administration [30,31,32,33]. The sodium–potassium ATPase hypothesis for BD was formulated by El-Mallakh and Wyatt in 1995 [34]. This concept was further confirmed in later molecular genetic studies [35,36]. Therefore, the mechanism of lithium action in this context could be related to the pathogenesis of bipolar disorder. Unfortunately, studies on the effect of lithium on Na and K-ATPase have not been continued in recent decades.

### 2.3. Second Messenger Systems

Another mechanism potentially involved in lithium’s modes of action is based on the second messenger systems. Second messenger systems modulate physiological processes by transmitting the signals from cell surface receptors (first messengers) to the intracellular effectors—enzymes, ion channels, and transporters. There are two major classes: cyclic nucleotide and phosphatidylinositol (PI) systems [37]. Both transpired to be connected with the pathogenesis of BD or/and lithium mechanisms.

#### 2.3.1. Cyclic Nucleotide System

A synthesis of messenger cyclic monophosphate adenosine (cAMP) as a result of epinephrine-caused adenylyl cyclase stimulation was described by Earl Sutherland (1915–1974), a Nobel laureate in medicine in 1971 [38]. Further studies showed that G-protein subunits are signal transducers between receptors and adenylate cyclase [39]. The research team led by Robert Haim Belmaker showed that lithium treatment inhibits the adenyl cyclase activity and reduces the cAMP accumulation in the brain [40]. Chronic lithium treatment was also shown to interfere with the dissociation of the G-protein subunit Gi [41]. Furthermore, Harvey et al. [42] showed that chronic lithium treatment resulted in decreased cAMP levels caused by the increased activity of cAMP-phosphodiesterase and increased cGMP levels in rat’s cortical regions.

In recent decades, evidence for the pathogenic role of adenylate cyclase in BD and the therapeutic mechanism of lithium was collected. In 2009, it was shown that lithium and carbamazepine preferentially inhibit specific isoforms—AC5, which might be connected with their antidepressant effect [43]. The genome-wide association study (GWAS) from 2014 revealed the adenylate cyclase-2 gene (*ADCY2*) as a risk gene for BD [44]. In 2020, Iranian researchers confirmed the association of the *ADCY2* polymorphism with BD and suggested it as a potential predictive marker of the lithium treatment response [45].

#### 2.3.2. Phosphatidylinositol System

The PI system is crucial for receptor-mediated signal transduction. It involves the hydrolysis of phosphoinositides and the release of inositol trisphosphate (IP3) as a second messenger. The lithium effect on the PI system resulting in the uncompetitive inhibition of inositol monophosphatase (IMPase) was first demonstrated by American researchers from St Louis, Loretta Hallcher and William Sherman, in 1980 [46]. In 1989, the British physiologist and biochemist Michael Berridge (1938–2020) described the role of inositol trisphosphate (IP3) in cell signaling and calcium regulation [47]. He also further proved the uncompetitive inhibitory mechanism of lithium on inositol phosphate metabolism and formulated an inositol depletion hypothesis of BD. According to this hypothesis, lithium influences the PI system by weakening the second signaling. This proposed lithium mode of action includes the mentioned inhibition of the IMPase, which leads to increased inositol-1-monophosphate (I1P) levels, followed by phosphoinositols accumulation, decreased phosphatidylinositol concentration, and altered levels of key membrane phospholipids [47].

Lithium also lowers the phosphatidylinositol/phosphatidylcholine ratio in cell membranes [48]; decreases the amount of phosphatidylinositol-4,5-bisphosphate (PIP2) levels in the BD patient’s platelet membrane; and reduces the phosphatidylinositol-specific phospholipase C (PIPLC), which is crucial for the generation of IP3, diacylglycerol, and also cAMP, resulting in the downregulation of inositol phosphates [49,50]. Israeli authors performed experimental studies on mice, where they deprived them of one of the two genes associated with the PI system: *IMPA1* (inositol monophosphatase-1) and *SMIT1* (sodium myo-inositol transporter-1). The study showed that both the genetic knock-out procedures caused behavior that resembled the effect after lithium administration, which may indicate that both inositol depletion and accumulation of phosphoinositols play an important role in the effects caused by lithium [51].

The 21st-century molecular–genetic studies suggest that the PI system may play a role in the pathogenesis of BD. A 2008 GWAS of BD patients identified an association with the di-acylglycerol kinase eTa (*DGKH*) gene, which encodes a crucial protein in the PI pathway [52]. In 2010, an association between polymorphism in the *IMPA2* gene and BD was found [53].

### 2.4. Glycogen Synthase Kinase-3 (GSK3) Activity

Glycogen synthase kinase-3 (GSK3) is a serine/threonine kinase that exists in two isoforms: GSK3 alpha (GSK3α) and GSK3 beta (GSK3β). It is involved in numerous signaling pathways, with over a hundred known substrates and multiple physiological functions. The GSK3β is connected, among others, with neurogenesis, neuronal polarization, axon growth, and biological rhythms [54].

The GSK3β is one of the best described molecular targets of lithium. In 1996, two independent studies by Peter Klein and Douglas Melton from the University of Pennsylvania [55] and Vuk Stambolic et al. from the University of Toronto [56] presented lithium’s direct competitive mechanism of GSK3β inhibition. Also, the indirect method of lithium-caused inhibition was described as involving the phosphorylation of N-terminal serines of both GSK3α and GSK3β.

In a recent review, the four most important substrates of GSK3β were indicated as potentially contributing to the lithium effects in BD. They include the transcription factor cAMP response element-binding protein (CREB), the RNA-binding protein FXR1 (Fragile X related protein 1), kinesin subunits, and the cytoskeletal regulator CRMP2 (Collapsin response mediator protein 2). All these substrates are, in different ways, associated with the pathogenesis of BD [57]. Lithium’s inhibition of GSK3β also influences the PI3K/Akt pathway, which leads to increased Akt-1 activity by inhibiting phosphoinositide 3-kinase-mediated Akt phosphorylation [58].

The next subsections show how many mechanisms of lithium can eventually be related to the inhibition of GSK3β. However, given the multiple substrates of this enzyme, it is hard to say which is most important to the drug’s therapeutic effect in BD. In a recent GWAS that involved 40,000 BD subjects, no common variants of the *GSK3β* gene were associated with an increased risk for BD [59], whereas it was found that the lithium effect on GSK3β might play a role in lithium-induced renal decline [60] or in the anti-suicidal properties of this ion [61].

### 2.5. Brain-Derived Neurotrophic Factor

The brain-derived neurotrophic factor (BDNF) was identified in 1982 and quickly became the most explored neurotrophin in studies on the pathogenesis and treatment of mental disorders [62]. BDNF is essential for the function and survival of neurons and the regulation of neurotransmitters, such as glutamate, gamma-aminobutyric acid, dopamine, and serotonin. The BDNF system is important for the pathogenesis and treatment of mood disorders [63]. The Val66Met polymorphism of the *BDNF* gene is associated with a predilection to the BD illness [64].

In experimental studies, lithium enhanced the BDNF expression in the brain. One of the mechanisms behind this might be the stimulation of CREB, which further activates the *BDNF* gene expression [65]. Clinical studies showed that BDNF serum concentrations are reduced during episodes of both mania and depression and increase after successful pharmacological treatment, including lithium treatment. A low concentration of BDNF is also regarded as a marker of late-stage bipolar affective disorder [66]. However, our previous study from 2010 found that in the group of excellent lithium responders, despite the long duration of BD (20 years or more), the serum BDNF was not different from healthy subjects [67]. This supports the previous evidence regarding lithium’s effect on BDNF expression and suggests that it restores the proper BDNF level. We also demonstrated that the Val allele of the BDNF Val66Met polymorphism in the BD patients’ group determines better cognitive functions associated with the prefrontal cortex activity. This phenomenon was specific to bipolar disorder and was not observed in schizophrenic patients or healthy subjects [68]. Our previous study also showed the prophylactic potential of lithium treatment is connected with the Val66Met polymorphism of the *BDNF* [69].

### 2.6. Neurotransmitters

Since the 1960s, neurotransmitters, such as catecholamines (norepinephrine, dopamine) and serotonin, have been implicated in the pathogenesis of mood disorders and the therapeutic mechanism of antipsychotic and antidepressant drugs [70,71,72]. The effect of lithium on these neurotransmitters was also investigated.

#### 2.6.1. Dopamine

In 2017, the latest variant of the dopamine hypothesis of BD was presented [73]. Hyperdopaminergia (elevations in D2/D3 receptor availability) was proposed to underline manic episodes, while increased dopamine transporter (DAT), resulting in reduced dopaminergic function, was suggested for depressive episodes. Since the 1970s, there has been evidence that lithium reduces dopaminergic activity, which may be connected with its antimanic effect [74]. Can et al. [75] demonstrated that chronic lithium treatment restored abnormal dopamine release in the nucleus accumbens. More recent reviews point to the regulation of dopamine transmission by lithium and the relationship of this effect to the lithium influence on GSK3β [76,77].

#### 2.6.2. Serotonin

A hypothesis for the mechanism of long-term lithium action by stimulating serotonergic neurotransmission was proposed forty years ago [78]. The review of Price et al. [79] suggests that lithium’s primary action on the serotonergic system may be presynaptic, with many postsynaptic effects leading to the enhancing effect on this neurotransmission. Recently, a study using positron emission tomography (PET) was performed with BD patients during a depressive episode. The patients with lower binding of the serotonin transporter and serotonin 5-HT1A receptor at the baseline showed more significant clinical improvement after the lithium treatment [80].

## 3. New Findings

### 3.1. In Vitro Studies

Research on lithium’s mechanisms in recent years applied novel in vitro methods using peripheral blood mononuclear cells (PBMCs), lymphoblastoid cell lines (LCLs), and induced pluripotent stem cells (iPSCs). In most studies, the characteristics of cells obtained from so-called “lithium responders” (LRs) and “lithium non-responders” (LNRs) were compared.

#### 3.1.1. Peripheral Blood Mononuclear Cells (PBMCs)

The study of human PBMCs from BD patients confirmed that lithium treatment results in the GSK-3β phosphorylation in Ser9, which can be evaluated as a biochemical marker of the therapeutic response to the drug [81]. It was also shown that PBMCs from BD patients and controls differ in immunophenotypes, showing immunologic imbalance in BD. The in vitro lithium treatment of PBMCs resulted in an increased percentage of CD14+ monocytes [82].

#### 3.1.2. Lymphoblastoid Cell Lines (LCLs)

Lymphoblastoid cell lines (LCLs) are cell lines derived from B lymphocytes, typically immortalized using the Epstein–Barr virus (EBV) [83]. It was found that lithium did not alter the gene expression in the LCLs of healthy subjects but significantly changed the expression in the LCLs of BD patients—mostly the genes involved in apoptosis [84]. In an LCL-based genome-wide expression study, 2060 genes were differentially expressed between LR and LNR after in vitro lithium treatment. The further pathways analysis and qPCR validation showed insulin-like growth factor 1 as a significant biomarker of the lithium response [85]. Another study showed that the lithium treatment of LCLs from LR restored the cell viability and influenced the GSK-3β expression [86]. When comparing the RNA of LCLs from BD females, greater expressions of the *HDGFRP3* (hepatoma-derived growth factor-related protein-3) and *ID2* (inhibitor of DNA binding 2) genes were found in the LRs. These genes are connected with neurogenesis and neurotrophic processes [87]. The immunoglobulin-related genes in LCLs differed between BD patients and control subjects and between the LRs and LNRs [88].

Lithium in vitro treatment’s effect on microRNA (miRNA) expression identified that seven miRNAs significantly changed after four days of treatment and four changed after 16 days of treatment, suggesting miRNAs as candidate’s biomarker of lithium treatment [89]. Another study identified the differences in 335 mRNAs and 77 miRNAs between LRs and LNRs, mainly related to the immune processes, suggesting lithium’s anti-inflammatory effects [90].

#### 3.1.3. Pluripotent Stem Cells

Pluripotent stem cells (PSCs) are a type of cells characterized by the ability to self-renew and differentiate into any kind of cells found derived from the inner cell mass of embryos, primordial germ cells, teratocarcinoma, or male germ cells [91]. The studies of iPSC neurons derived from BD patients showed reversed hyperexcitability after the treatment only in neurons from LRs [92,93]. The cortical spheroids, or brain-like tissues derived from the iPSCs of BD patients, exhibited a smaller size and lower neuron excitability of the neurons compared with the healthy subjects. Treatment with lithium resulted in the normalization of the excitability of neurons to levels seen in controls [94]. The refurbishment of neuronal hyperactivity in LR was related to sodium currents and the Akt signaling pathway and to protein kinase B, which is connected with the growth and proliferation of the cell [95]. In LRs, neuronal progenitor cells derived from iPSCs demonstrated a high resistance to methamphetamine’s toxic action [96].

On the other hand, in LNRs, a reduced expression of the *LEF1* gene (lymphoid enhancer-binding factor 1), which modulates neuronal activity, was shown [97]. Research on iPSC-derived cortical neurons corroborated the role of the PI system in lithium mechanisms. Inhibition by lithium of the IMPA1 enzyme, a crucial PI system element, was associated with reducing the neuronal excitability and releasing calcium ions [98].

### 3.2. Biological Rhythms

Circadian rhythms are intrinsic, 24 h cycles that regulates various physiological processes, including the sleep–wake cycle, body temperature regulation, and hormone secretion, and is driven by molecular clocks and synchronized with the external environment, primarily through light–dark cycles [99]. They are regulated by clock genes constituting feedback loops of transcription and translation. The positive feedback loop includes the aryl hydrocarbon receptor nuclear translocator-like (ARNTL) (also known as BMAL1) and circadian locomotor output cycles kaput (CLOCK). The CLOCK-BMAL1 complexes activate the expression of the nuclear receptors REV-ERBα and RORα and several clock genes, such as period (PER1/PER2) and cryptochrome (CRY1/CRY2) [100]. The animal model of bipolar mania is also based on *Clock*Δ19 mice with a disrupted molecular circadian clock [101]. Interestingly, clinical research on BD patients revealed characteristics of biological rhythm disruptions, with a predominance of the eveningness chronotype [102]. The results from 95 studies showed that lithium delays the phase in various circadian rhythms (sleep–wakefulness cycles, activity rhythms, and peaks in elevation of body temperature) and also lengthens the circadian period [103]. The research on skin fibroblasts showed that LR patients more frequently exhibit the morningness chronotype [104] and that lithium treatment normalized circadian rhythm disturbances [105]. Corresponding results were shown in the study from research centers in Poznan and Cracow, Poland, where lithium-treated BD patients more often presented the morningness chronotype [106]. In the recent study by Mishra et al. [107], authors using a fibroblast-based model demonstrated a differential expression of circadian clock genes both between patients with BD and control persons, as well as between LRs and LNRs, with the meaningful distinction in the *Per 1* (clock protein PERIOD 1) and *Per 3* genes. Our study showed the connection with the effect of lithium prophylaxis was observed for six SNPs (single nucleotide polymorphisms) and three haplotype blocks of the *ARNTL* gene and two SNPs and one haplotype block of the *TIM* (timeless) gene [108].

The nuclear receptor REV-ERBα was also shown as a potential target for GSK3β and lithium [109]. In the mouse-based study, lithium-treated tissue slices and cells showed the elongation of period locomotor activity rhythms; lengthening of the molecular oscillations in the suprachiasmatic nucleus, lung tissue, and fibroblasts; and elevating *Per2* expression. The described effects might be caused by the lithium effect on GSK3β [110].

### 3.3. Telomere Functions

A protective cap protects the ends of chromosomes. This structure is called telomere (from the Greek words *telos*—end and *meros*—part), and its protective function is telomere capping. The 2009 Nobel Prize winners Elizabeth Blackburn, Carol Greider, and Jack Szostak explained the nature of telomeres and showed the mechanism behind chromosome protection by telomeres and the role of telomerase [111].

The telomere length can be regarded as a trait of aging processes. GWASs showed the genetic influence behind this effect, with the essential contribution of the leucine-rich repeat gene (*LRRC34*) found [112]. Shorter telomeres were also observed in patients with BD, and their length was normalized after treatment with lithium [113,114,115]. It was also discovered that the duration of lithium treatment is positively associated with the leukocyte telomere length in BD patients [116] and positively correlated with the expression of the telomerase reverse transcriptase (TERT) enzyme [117]. On the other hand, the recent research, including 591 lithium-treated patients, did not reveal a relationship between the time of lithium administration and the length of telomeres [118].

Interestingly, the study on an animal model of depression showed that a lithium-naïve Flinders Sensitive Line rats presented with a shorter telomere length, downregulated telomerase reverse transcriptase expression, and reduced telomerase activity. Lithium treatment restored the telomerase activity and normalized the telomerase reverse transcriptase expression in the rat’s hippocampus. This result might suggest a protective effect against telomere shortening and the neuroprotective role of lithium [119].

### 3.4. Immunomodulatory Effects

Granulopoiesis induction is a long-known effect of lithium treatment on the bone marrow due to increasing the granulocyte colony-stimulating factor [120,121]. Recent studies highlighted the immunomodulatory effect of lithium in the context of low-grade chronic inflammation and the microglial hypothesis of BD [122].

In a recent review by Sakrajda and Szczepankiewicz [123], evidence is presented for lithium’s reduction of proinflammatory cytokines, i.e., Interleukin (IL)-1β, Interferon (IFN)-γ, Tumor Necrosis Factor (TNF), IL-6, and Monocyte Chemoattractant Protein (MCP)-1 expression, as well as the elevated expression of the anti-inflammatory cytokine IL-10. Lithium’s action on the inflammatory system might be related to the lithium inhibition of GSK-3β. The GSK-3β activation is an important regulative mechanism of the NOD-like receptor protein 3 (NLRP3) inflammasome activation, which affects the production of inflammatory cytokines [124]. We previously showed that the mood-stabilizing treatment (with lithium or other drugs) of BD patients influences the inflammasome-regulated cytokines in euthymic BD patients. Still, only lithium-treated patients presented elevated expression of IL-10 [125]. Similar results were presented in the most recent review by Odeya Damri and Galila Agam [126], showing that chronic lithium treatment leads to altered cytokines expression in BD patients, decreases the secretion of inflammatory mediators in peripheral blood leukocytes, and also reduces the secretion of IL-1β in monocytes in vitro. Interestingly, the study by Sakrajda et al. [127], which used the in vitro model of neuroinflammation with the microglia cell line (HMC3) and focused on NLRP3 inflammasome involvement in BD, showed that lithium treatment with concentrations similar to those observed in the brains (0.5 mM) with microglia induced with innate-immune cytokines caused a significant anti-inflammatory effect mediated via GSK-3β inhibition, which affected NLRP3 inflammasome priming; its activation; and as a result, IL-1β protein secretion [127].

### 3.5. Antiviral Effects

The lithium immune-related mechanism also includes its antiviral actions. The first description was made by Julian Lieb in 1979, presenting two patients with recurrent herpes virus infections that went into remission during lithium treatment [128]. One year later, researchers from the University of Birmingham, using a hamster kidney model, demonstrated lithium’s inhibition of the herpes simplex virus (HSV) [129].

A retrospective assessment of labial herpes recurrences in patients receiving prophylactic lithium was performed in joint research between the Department of Adult Psychiatry, Poznan University of Medical Sciences, and the Department of Psychiatry of the University of Pennsylvania. In the group of Polish patients, over the course of the lithium administration, the termination of herpes recurrences took place in 46% of patients with labial herpes and the overall reduction in recurrence incidence was 64%. The more pronounced effect was revealed in subjects with serum lithium concentrations > 0.65 mmol/L and red blood cell lithium concentrations >0.35 mmol/L. In the group of American lithium-treated BD patients, the recurrences of labial herpes, compared with the five-year pre-lithium period, were reduced by 73%. On the other hand, patients with recurrent depression that received antidepressant drugs showed no difference in the recurrences of herpes compared with the five-year pretreatment period [130].

The anti-herpes effect of lithium could be related to its prophylactic and therapeutic effect in dementia, as the infection with herpes simplex virus type 1 (HSV1) produces a significant risk factor for Alzheimer’s disease [131].

The research on lithium and RNA viruses is concerned mainly with respiratory infections. In a retrospective analysis, a significant decrease in the average yearly rates of flu-like infections was found in patients that took lithium but not those that received antidepressants [130]. Also, a 28% decrease in respiratory infections, possibly partly due to the RNA viruses, was observed during long-term treatment with lithium [131].

Shortly after the outbreak of the COVID-19 epidemic, the antiviral potential of lithium on coronaviruses shown in experimental studies was reviewed [132]. An increasing hope was expressed that lithium could be useable in the anti-COVID-19 treatment [133]. Only one randomized clinical study found that lithium augmentation could reduce the severity of the COVID-19 course [134]. Moreover, the review of electronic records of 26,554 patients demonstrated that the frequency of infection was lower in subjects with therapeutic lithium levels (0.5–1.0 mmol/L) compared with those with lithium concentrations < 0.5 mmol/L [135].

### 3.6. Effect on Mitochondria

The upregulated production of oxidative stress markers leading to mitochondrial, dopamine system, and immune system dysfunction has been described in BD patients [136,137]. Lithium has been described as a modulator of the electron transport chains (ETCs) and oxidative phosphorylation (OXPHOS) pathway activity, enhancing oxidative phosphorylation in the frontal cortex of postmortem BD patients [138]. The in vitro study based on human neurons (SH-SY5Y) and chronic lithium treatment resulted in increased resistance to oxidative stress and enhanced glucose consumption and glycolysis activity [139]. Furthermore, a clinical study reported that lithium treatment increased the complex I activity and enhanced OXPHOS, positively correlating with plasma lithium levels in BD patients [140]. The 2018 study by Stacey et al. [141] presented the co-expression network analysis of RNA-seq data from lithium-treated BD patients, with an over-representation of genes involved in mitochondrial functioning (ETC and OXPHOS-related genes) and also the downregulation of these genes’ expressions in LRs compared with poor lithium responders. Interestingly, the previously described lithium’s inhibitory effect on the GSK-3β might also be involved in its influence on mitochondria, as this kinase was shown to interact, i.a., with mitochondrial proteins and respiratory chains and regulate the various mitochondrial functions [142].

### 3.7. Genetic Studies

In the 21st century, research emerged that explored the genetic foundations of lithium’s therapeutic mechanisms and efficacy, where various methodologies were employed. In most studies, the lithium response was measured using the Retrospective Assessment of the Lithium Response Phenotype Scale, known as the Alda Scale [143].

#### 3.7.1. Candidate Genes

In 2013, a review of candidate genes connected with lithium’s prophylactic response appeared, written by one of the authors of this review [144]. Since then, some additions were also performed in the Poznan center that, e.g., showed the association of lithium efficacy with stress response genes [145]. In 2021, Senner et al. [146] found candidate genes that showed an association with lithium efficacy, where at least two studies listed six genes, namely, *GSK3β*, *BDNF*, serotonin transporter (*5HTTLPR*), calcium voltage-gated channel auxiliary subunit gamma 2 (*CACNG2*), nuclear receptor subfamily 1 group D member 1 (*NR1D1*), and dopamine receptor D1 (*DRD1*) genes.

#### 3.7.2. Genome-Wide Association Studies (GWASs)

The first GWAS of the lithium response in patients with BD was performed by Perlis et al. in 2009 [147]. They assessed the five-year risk of recurrence in 1177 BD patients, including 458 treated with lithium. When comparing the LRs with healthy controls, a difference was found in the *GRIA2* gene, which codes the ionotropic AMPA glutamatergic receptor. In 2014, Chen et al. [148] investigated the population with Han Chinese ancestry and demonstrated the association of the lithium response with the gene encoding glutamate decarboxylase-like protein 1 (*GADL1*). Two years later, Song et al. [149], on a sample of 3874 lithium users from Sweden and the UK, showed an association between the lithium response and the *SEC14* and spectrin domains 1 (*SESTD1*) genes, which encode a synapse protein that directly binds to phospholipids. A GWAS was also performed, which recognized a region on chromosome 3, p21.1, with the gene *GNL3* (G protein nucleolar 3), as essential for the proliferation of neuronal progenitor cells by lithium [143].

#### 3.7.3. ConLiGen Project

The Consortium of Lithium Genetics (ConLiGen) was established in 2009 as a collaborative initiative by the International Group for Study of Lithium-treated Patients (IGSLI) and the National Institute of Mental Health. The initial report about this event was published in 2010 [150]. The first results of a GWAS that concerned molecular–genetic factors connected with the preventive effect of lithium and involved 2563 patients from 22 sites were reported six years later. The association of the lithium effect was found with a region on chromosome 21, including two long non-coding RNAs (lncRNAs) that modulate the central nervous system’s expression of the genes [151]. Subsequently, ConLiGen tried to assess the association between lithium efficacy in BD and the polygenic risk score (PRS) for different diseases. It turned out that the PRS characteristic for schizophrenia was linked to a poorer lithium effect [152], which correlated with research performed in Poznan, pointing to a negative connection between lithium’s prophylactic effect and a predisposition to psychotic symptoms [153]. Negatively connected with lithium efficacy were also the PRS for major depressive disorder [154] and the PRS for attention deficit hyperactivity disorder (ADHD) [155]. A worse response to lithium was also associated with a higher PRS for diabetes and hypertension in bipolar 1 but not bipolar 2 patients [156].

The ConLiGen also assessed the relationship of lithium’s prophylactic effect to human leukocyte antigen (HLA) genes. It turned out that a positive lithium effect was connected with HLA genes for decreased inflammation, while a negative effect was associated with HLA genes for increased inflammation [157]. Moreover, Amare et al. [152] recognized 36 candidate genes mainly connected with the cholinergic and glutamatergic systems, linked to a positive lithium effect. Recently, a collaborative study between the groups of ConLiGen and Pharmacogenomics of Bipolar Disorder demonstrated an association between lithium prophylaxis with focal adhesion and the PI3K-Akt signaling pathway, which are connected, i.a., with the proliferation and growth of the cell [158].

In 2023, ConLiGen’s work based on a comprehensive analysis of genetic and clinical data from 2367 BD patients treated with lithium proposed the polygenic score for lithium treatment response (Li^+^_PGS_) [159]. The proposed biological marker showed statistically significant associations with the lithium treatment response, and Li^+^_PGS_ might be a helpful pharmacogenomic tool to predict the patient’s response to lithium therapy in the future.

#### 3.7.4. Epigenetic Findings

The epigenetic studies of French researchers identified the biomarkers connected with the efficacy of lithium. The methylation differences between LRs and LNRs were disclosed using a genome-wide methylomic approach (SeqCapEpi) [160]. The combination of epigenetic factors with clinical ones, such as the first episode polarity, psychotic symptoms, and BD family load, made it possible to predict the effect of lithium for 86% of patients [161]. The confirmation of specific methylation patterns determining the lithium response in BD also came from a recent study that obtained 130 differentially methylated positions and 16 differentially methylated regions in LRs vs. LNRs [162].

## 4. Conclusions

Although certainly not complete, this review shows that the possible mechanisms of lithium action are multifold and still interesting research topics. In this review, the evolution of the views on these mechanisms starting in the mid-19th century is shown, while trying to distinguish between old and new findings. However, it turns out that many apparently “old” mechanisms had their revival in research performed in the 21st century. We also aimed to signal the potential links and overlaps between the proposed mechanisms of action, which might explain the widely described multimodality of lithium action. Notably, many eventually converged toward the lithium inhibition of GSK-3β and PI, the mechanisms postulated in the 1980s/1990s. In 2013, Brown and Tracy assumed these mechanisms to be the most important for lithium action at the cellular level [163], and in 2016, Malhi and Outhred simply stated that the research in the 21st century cemented GSK-3β inhibition as a key mechanism underpinning the effects of lithium [164]. In a recent review of the biological bases and treatment strategies in BD, the inhibition of the PI system and GSK-3β as the main mechanisms for lithium action are also underscored [165].

We also want to emphasize the high heterogeneity of the studies’ results on different mechanisms mentioned in this review. In our opinion, this might have resulted from several factors, including the different study designs used, differences between the studied populations and between each patient, lack of longitudinal studies that observed the changes in the same subjects during chronic treatment, or differences in patient treatment.

While some of the lithium mechanisms discussed in our review are linked to the pathogenesis of BD, many of these relationships are, at best, tentative. On the other hand, certain findings associated with these mechanisms could serve as markers for the lithium response, and several are proposed as such. Advances in molecular genetics may enable the inclusion of genetic markers for this purpose, which was recently described in studies from ConLiGen, although the application of genetic markers in clinical practice may take time. However, based on lithium’s multimodal action and the beneficial effect of good responsiveness to treatment, such integration would be invaluable for identifying the best candidates for long-term lithium therapy, which is the most effective method of BD prophylaxis, though it has recently been underutilized.

The main directions for repurposing lithium beyond BD are the augmentation of antidepressants in treatment-resistant depression [4] and the use in neurodegenerative disorders, such as Alzheimer’s disease [166]. Interestingly, the effect of lithium augmentation of antidepressants was previously associated with a polymorphism of the *GSK-3β* gene [167]. In Alzheimer’s disease, the mechanisms of lithium action may also involve the GSK-3β inhibition [168], lithium’s effect on mitochondria [169], and its anti-herpes activity [129,131].

Therefore, it might be concluded that despite gathering substantial information on this subject, further studies are still necessary to understand lithium’s modes of action.
